# Auxin and Cell Wall Invertase Related Signaling during Rice Grain Development

**DOI:** 10.3390/plants3010095

**Published:** 2014-02-07

**Authors:** Sarah Russell French, Yousef Abu-Zaitoon, Md. Myn Uddin, Karina Bennett, Heather M. Nonhebel

**Affiliations:** 1 Molecular and Cellular Biology, University of New England, Armidale, New South Wales 2351, Australia; E-Mails: SKRussellFrench@dow.com (S.R.F.); kbennett@peracto.com (K.B.); 2 Department of Biology, Faculty of Science, Islamic University, Madinah 170, Saudi Arabia; E-Mail: yousefaz@yahoo.com; 3 Department of Microbiology, University of Chittagong, Chittagong 4331, Bangladesh; E-Mail: mynuddincu@gmail.com

**Keywords:** grain-fill, rice, indole-3-acetic acid, cell wall invertase, invertase inhibitor, OsIAA29, OsHAP3D, ZmEBE-like, lipid transfer protein, defensin-like

## Abstract

Indole-3-acetic acid (IAA) synthesis is required for grain-fill in maize and appears to be regulated by cell-wall invertase (CWIN) activity. *OsYUC12* is one of three IAA biosynthesis genes we previously reported as expressed during early rice grain development, correlating with a large increase in IAA content of the grain. This work aimed to investigate further the role of *OsYUC12* and its relationship to CWIN activity and invertase inhibitors (INVINH). The analysis shows a brief peak of *OsYUC12* expression early in endosperm development. Meta-analysis of microarray data, confirmed by quantitative expression analysis, revealed that *OsYUC12* is coexpressed with *OsIAA29*, which encodes an unusual AUX/IAA transcription factor previously reported as poorly expressed. Maximum expression of *OsYUC12* and *OsIAA29* coincided with maximum CWIN activity, but also with a peak in *INVINH* expression. Unlike *ZmYUC1*, *OsYUC12* expression is not reduced in the rice CWIN mutant, *gif1*. Several reports have investigated *CWIN* expression in rice grains but none has reported on expression of *INVINH* in this species. We show that rice has 54 genes encoding putative invertase/pectin methylesterase inhibitors, seven of which are expressed exclusively during grain development. Our results suggest a more complex relationship between IAA, CWIN, and INVINH than previously proposed.

## 1. Introduction

Several recent publications indicate key intersecting signaling roles for indole-3-acetic acid (IAA) and cell wall invertases (CWIN) during cereal grain development. The maize *defective endosperm18* (*de18*) phenotype was recently shown to result from loss of expression of the IAA biosynthesis gene *ZmYUC1* and related low levels of IAA in the developing grain [1]. The cell wall invertase *miniature1* (*mn1*) mutant also shows poor grain fill, the low levels of cell wall invertase resulting in a defective basal endosperm transfer layer (BETL) with poorly developed wall in-growths [2]. Maize *mn1* mutants have low levels of IAA and low expression of *ZmYUC1*; glucose was able to increase *ZmYUC1* transcript levels in cultured kernels [3]. Forestan *et al.* [4] showed that IAA accumulates in the BETL, aleurone and embryo surrounding region (ESR) just before the endosperm starts to accumulate starch. In addition, both BETL and ESR showed a high level of auxin transporter *ZmPIN1* transcript and protein. The rice ortholog of *Mn1*, *GIF1/OsCIN2*, also appears to be important for grain development, with *gif1* mutants (that have much lower CWIN activity in developing grains) showing poor grain-fill; expression of *GIF1* during grain development is localized to the vascular trace [5]. The relationship between IAA and invertase in rice has not been investigated. 

In a previous paper on IAA synthesis in developing rice grains we showed that IAA accumulates more than 50-fold in developing kernels during endosperm cellularisation and early starch deposition [6]. The rise in IAA content was correlated with a large increase in expression of IAA biosynthesis genes *OsYUC9*, *OsYUC11*, and *OsTAR1*. We also obtained some rather inconsistent data on a third endosperm-specific YUCCA gene, *OsYUC12*. Preliminary results suggested that *OsYUC12* was expressed only briefly during grain development and may have a distinct role from the more highly expressed *OsYUC9* and *OsYUC11*. The aim of the work reported here was to clarify the expression of *OsYUC12* in developing grains and to investigate its relationship to CWIN, as well as the unexpected observation that *OsYUC12* is coexpressed with a putative invertase inhibitor (INVINH). 

A number of reports, e.g., Jin *et al.* [7], indicate that invertases in dicots are regulated by INVINH. In addition, ZM-INVINH1 inhibited maize CWIN activity *in vitro*, bound to a glycoprotein fraction including CWIN, and was localized to the ESR of young developing maize kernels [8]. Several papers report on expression of *CWIN* in rice, e.g., [9], however the role of invertase inhibitors in rice has been neglected. Furthermore, extraction of invertase for assay of its activity has normally been carried out at pH 7.5, e.g., [10], conditions under which any bound inhibitor would be expected to dissociate from the enzyme [11]. In this work, we carried out a comprehensive phylogenetic analysis of invertase inhibitor homologs in rice; we investigated the expression of Os04g49720, a co-ortholog of *ZM-INVINH1* previously designated as *OsINVINH3* [8]; we extracted and assayed CWIN activity under conditions that have been shown to preserve an enzyme/inhibitor complex [8]. Our data on expression of *OsYUC12* and *OsINVINH3*, as well as CWIN activity suggest that the relationship between IAA and CWIN is more complex than previously proposed. 

In addition, preliminary work suggested a second auxin related gene, *OsIAA29*, encoding a putative AUX/IAA transcription factor is coexpressed with *OsYUC12* and *OsINVINH3*. Coexpression analysis has been used productively to predict networks of genes that may act together in a common pathway, e.g., [12]. To explore other genes that may act in the same signaling pathway as *OsYUC12*, we carried out a comprehensive coexpression meta-analysis using *OsYUC12*, *OsIAA29*, and *OsINVINH3* as bait genes. The search for coexpressed genes revealed a number of sequences encoding uncharacterized proteins, some of which are homologs of proteins with an identified signaling role earlier in plant reproductive development. In addition, a number of the coexpressed genes encode homologs of molecules specifically found in endosperm transfer cells of other cereals. We suggest that the coexpressed genes may encode novel signaling factors.

## 2. Results

### 2.1. OsYUC12 and Other Endosperm YUCCA Genes Are Conserved in Cereal Species

In preparation for investigating further the role of *OsYUC12* in grain-fill we asked the question whether this gene had conserved orthologs in other cereal species. A phylogenetic analysis of *OsYUC9*, *OsYUC11*, and *OsYUC12* in maize, sorghum, and rice (Supplemental Figure S1) showed that each of these YUCCA genes has clear orthologs in each species. Secondly, we explored the expression of *GRMZM2G107761_T01*, the maize ortholog of *OsYUC12*, using microarray data from Sekhon *et al.* [13] accessed via PLEXdb [14] as experiment ZM37. This revealed that *GRMZM2G107761_T01* was also expressed primarily during grain development with a peak at eight days after pollination (DAP) (Supplemental Figure S2). 

### 2.2. Expression of OsYUCCA Genes in the gif1 CWIN Mutant

**Figure 1:**
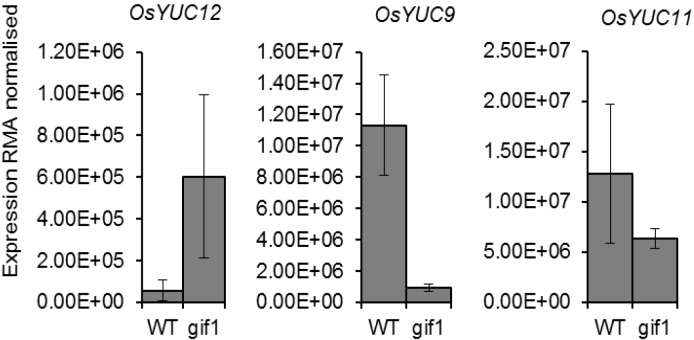
Comparison of expression of *OsYUC9*, *OsYUC11* and *OsYUC12* in developing grains (7 DAP) from the *gif1* mutant and wild type using RMA normalized microarray data from Wang *et al.* [5] “Global gene expression profiles of *Oryza sativa* wild type Zhonghua11 and mutant *gif*1 in filling stage” accessible as accession OS17 in PLEXdb [14]. Data represent the mean of three samples ± standard error.

In light of the low expression and activity of ZmYUC1 in the maize CWIN mutant *mn1*, we investigated expression of *OsYUC9*, *OsYUC11*, and *OsYUC12* in the equivalent *gif1* mutant of rice using data from Wang *et al.* [5] “Global gene expression profiles of *Oryza sativa* wild type Zhonghua11 and mutant *gif*1 in filling stage” accessible as accession OS17 in PLEXdb [14]. The data shown in Figure 1 indicates that expression of *OsYUC9* only is reduced in the *gif1* mutant. 

### 2.3. Invertase Inhibitor Phylogeny

**Figure 2:**
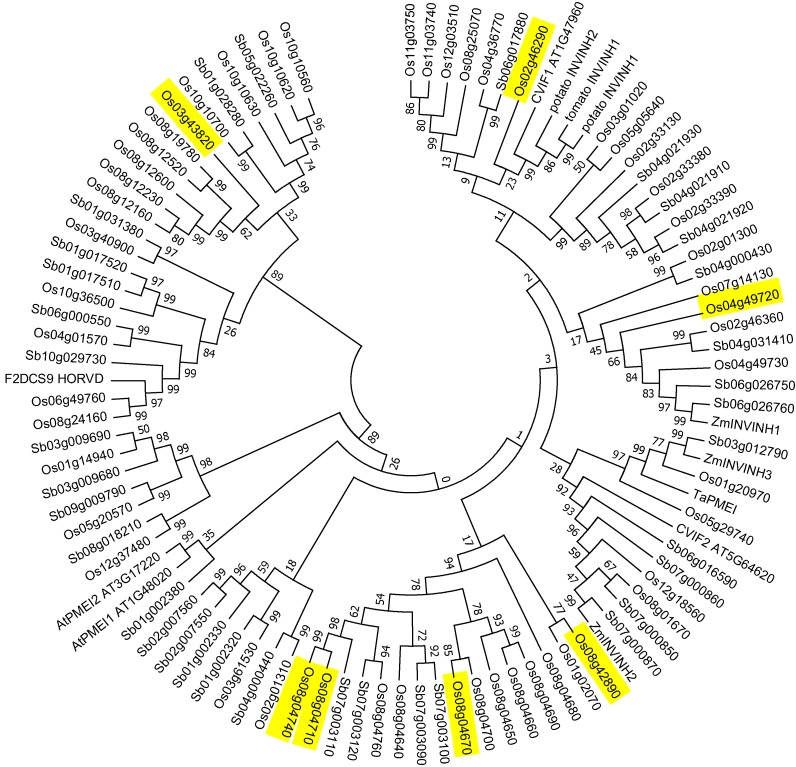
Phylogenetic tree showing relationships between INVINH and PMEI homologs from rice and Sorghum; protein sequences for ZM-INVINH1, ZM-INVINH2, ZM-INVINH3, and experimentally characterized PMEIs and INVINH from wheat, potato, tomato, and *Arabidopsis* are also included for comparison. The tree was produced in MEGA5.2 [25] using the Neighbor-Joining method [26]. MUSCLE [27] was used for multiple sequence alignment. The bootstrap consensus tree was inferred from 500 replicates [28]. Evolutionary distances were computed using the Poisson correction method [29]. All ambiguous positions were removed for each sequence pair. Highlighted sequences are encoded by genes expressed in endosperm during early caryopsis development.

A BlastP search of the rice proteome using query sequences ZM-INVINH1, ZM-INVINH2, and ZM-INVINH3 initially identified 20 homologous proteins. Additional rice homologs, as well as sequences from sorghum, were obtained using the homologous protein function in Phytozome. The phylogenetic tree shown in Figure 2 resulted from analysis of 54 protein sequences from rice, 34 sequences from sorghum, as well as experimentally characterized pectin methylesterase inhibitors (PMEIs) from wheat [15] and *Arabidopsis* [16], INVINHs from tomato [7], potato [17], and Arabidopsis [16] and F2DCS9 HORVD, an invertase inhibitor homolog from barley identified as up-regulated under heat stress by Mangelsen *et al.* [18]. The comprehensive set of homologs from the sorghum proteome was included to identify rice sequences that were conserved in more than one cereal species.

A number of bootstrap values for larger clades are low due to the highly diverse nature of the sequences. However, an examination of the multiple sequence alignment indicated that the four conserved cysteine residues previously shown to form disulfide bridges in PMEIs and INVINHs [19] were present in most sequences and were correctly aligned (Supplemental Figure S3). Branches with high bootstrap values indicate reliable prediction of phylogenetic relationships. These show that sorghum has unambiguous orthologs of ZM-INVINH1, ZM-INVINH2, and ZM-INVINH3. Rice also has clear orthologs of both ZM-INVINH1 (Os04g49730) and ZM-INVINH3 (Os01g20970). Further analysis of sequences most similar to ZM-INVINH1 indicated that the tandem repeats Os04g49730 and Os04g49720, as well as Os02g46360 are co-orthologous to ZM-INVINH1, the only characterized invertase inhibitor from a cereal [8]. Interestingly, characterized invertase inhibitors from dicot species are in separate clades from ZM-INVINH1. Arabidopsis invertase inhibitor, C/VIF1, as well as potato and tomato invertase inhibitors do not have any unambiguous cereal orthologs. On the other hand, C/VIF2 falls into a well-supported clade containing ZM-INVINH2, two rice sequences and four sorghum sequences. ZM-INVINH3 and Os01g20970 are orthologs of TaPMEI and are, therefore, likely to be PMEIs rather than invertase inhibitors. The Arabidopsis PMEIs appear in a separate branch from cereal PMEIs. The tree contains many orthologous pairs from rice and sorghum; however rice has many more sequences which appear to be due to recent gene duplication as evidenced by groups of tandem repeats, particularly on chromosome 8. The barley protein F2DCS9_HORVD, which has been given the name INVINH1 [18], is only distantly related to other characterized invertase inhibitors but has unambiguous orthologs in both rice and sorghum. 

### 2.4. Analysis of OsCIN2/GIF1 and Putative INVINH Expression in Developing Caryopses Using Published Microarray Data

The extracellular invertase GIF1/OsCIN2 has been shown to be responsible for most of the CWIN activity in developing rice grains [5]. We mined microarray data for information on expression of *GIF1* as well as the 54 invertase inhibitor homologs during grain development. Data was accessed via PLEXdb [14] from experiment accessions OS5 “Expression data for reproductive development in rice” [20], OS8 “Expression data from rice embryo, endosperm, root, leaf, and seedling” [21], OS16 “Genome-wide gene expression profiling of rice stigma” [22], OS44 “Rice expression atlas (3): Early embryogenesis” [23] and OS89 “Expression data from rice embryo and endosperm development” [24].

**Figure 3:**
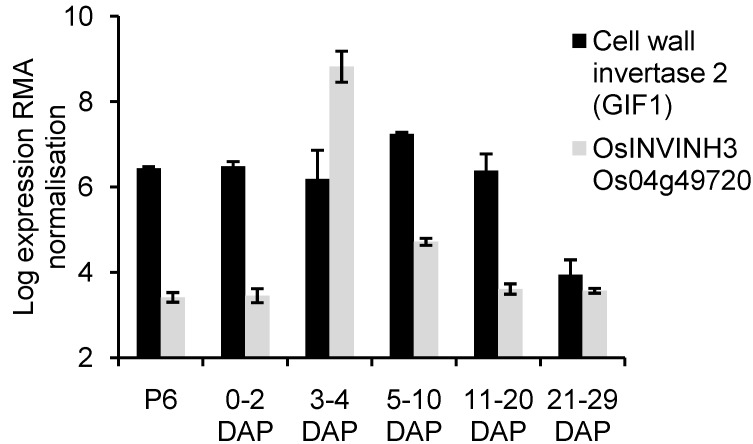
Comparison of expression of cell wall invertase *GIF1* (Os04g33740) and *OsINVINH3* (Os04g49720) during grain development using RMA normalized data from Expression data for reproductive development in rice [26] accessed as accession OS5 from PLEXdb [14]. Data represent the mean of three samples ± standard error. Data on expression of other cell wall invertases during grain development from the same source is provided as Supplemental Figure S4.

Seven *INVINH/PMEI* homologs (Table 1) were found to be expressed exclusively during caryopsis development with expression highest in samples three to four days after pollination (DAP); expression appeared specific to the endosperm. Caryopsis-expressed INVINH/PMEI homologs included Os04g49720 which is co-orthologous with Os04g49730 and Os02g46360 to ZM-INVINH1. Os04g49730 and Os02g46360 appeared to be only weakly expressed; Os04g49720 has previously been designated as OsINVINH3 [8]. In contrast to the large changes in expression of *OsINVINH3*, there was little change in the expression of *GIF1* from prior to anthesis until 20 DAP (Figure 3). Interestingly examination of endosperm-specific genes in Figure 2 showed them to be present in diverse clades. We also noted that the expression profiles of genes in Table 1 matched that of IAA biosynthesis gene *OsYUC12* and the gene encoding putative AUX/IAA transcriptional regulator OsIAA29. 

**Table 1:** Invertase inhibitor/pectin methylesterase inhibitor (INVINH/PMEI) homologs from rice identified as expressed during caryopsis development using microarray data accessed via PLEXdb [14] from experiment accessions OS5 “Expression data for reproductive development in rice” [26], OS8 “Expression data from rice embryo, endosperm, root, leaf, and seedling” [21], OS16 “Genome-wide gene expression profiling of rice stigma” [27], OS44 “Rice expression atlas (3): Early embryogenesis” [28], and OS89 “Expression data from rice embryo and endosperm development” [29].

Locus ID	Expression
Os02g46290.1	2 to 4 DAP, endosperm
Os03g43820.1	3-4 DAP, 5-10 DAP, endosperm
Os04g49720.1	3-4 DAP, 5-10 DAP, endosperm
Os08g04670.1	3-4 DAP, endosperm
Os08g04710.1	3-4 DAP, endosperm
Os08g04740.1	3-4 DAP, 5-10 DAP, endosperm
Os08g42890.1	3-4 DAP, 5-10 DAP, endosperm

### 2.5. Coexpression Meta-Analysis Using Bait Genes OsIAA29, OsYUC12 and OsINVINH3

Several on-line platforms are available for coexpression analysis including RiceNet [30], GeneCAT [31], and Rice Oligonucleotide Array Database [32]. Initial investigations indicated that the limited data set used in RiceNet led to the identification of some genes that had poorly correlated expression with our genes of interest in data sets, such as OS44 [28], which includes samples collected daily during the early stages after pollination. Rice Oligonucleotide Array Database contains more data sets but as most of these did not contain caryopsis tissue and the site does not allow for condition dependent selection of data sets, results were similarly unsatisfactory. We therefore downloaded RMA normalized datasets via PLEXdb for previously mentioned accessions OS5, OS8, OS16, OS44, and OS89 and calculated pairwise mutual coexpression ranks [33] for three bait genes: *OsIAA29*, *OsYUC12*, and *OsINVINH3*. Examination of the expression profiles of genes with a mutual co-expression rank <50 in individual experiments indicated very close correspondence with expression of bait genes. All appear to be expressed exclusively in the endosperm of developing grains with maximal expression at 3–4 DAP.

Genes with a mutual co-expression rank less than 20 (MR < 20) are shown in Table 2, with a larger set (MR < 50) shown in Supplemental Table S1. All database annotations have been manually checked against the literature; where the annotation differs from the literature, the published protein designation has been used. Table 2 includes two lipid transfer protein-like genes (*LTPL*) and four defensin/defensin like genes (*DEF/DEFL*). This group of genes encoding cysteine-rich peptides (CRPs) was also highly represented in the larger MR < 50 group, with a total of 13 genes present. Also present was a *ZmEBE-like* (embryo sac/basal endosperm transfer layer/embryo surrounding region) gene; a further four homologs of this gene are found in the MR <50 group. A third group of homologous proteins found in the MR <50 group included two additional INVINH/PMEI homologs. Table 2 also includes genes encoding OsHAP3D/OsLEC1A (Leafy cotyledon1) [34] and a leucine-rich repeat receptor-like kinase. Genes with a likely signaling role in the MR <50 group also include OsRR33, a type-B response regulator [35] an ortholog of OBERON1/2 [36] and at least two other proteins with a predicted nuclear location.

**Table 2:** Genes with a pairwise mutual coexpression rank < 20 with each of the bait genes, *INVINH3*, *OsIAA29*, and *OsYUC12*. Genes listed in order of increasing mutual co-expression rank.

Transcript ID	Description
Os12g13960.1	Lipid transfer protein-like LTPL33 [37]
Os02g07628.1	Defensin DEF5 [37]
Os11g45360.1	Defensin-like DEFL15 [37]
Os01g70680.1	Defensin DEF1 [37]
Os02g07624.1	Defensin DEF4 [37]
Os11g18140.1	homologous to ZmEBE 1 (embryo sac/basal endosperm transfer layer/embryo surrounding region [38]
Os06g17480.1	Nuclear factor YC OsHAP3D /OsLEC1A [34]
Os05g07850.1	Leucine-rich repeat receptor-like kinase [39]
Os11g14880.1	Lipid transfer protein-like LTPL32 [37]

### 2.6. Quantitative Expression Analysis of Genes Coexpressed with OsIAA29, OsYUC12, and OsINVINH3

**Figure 4:**
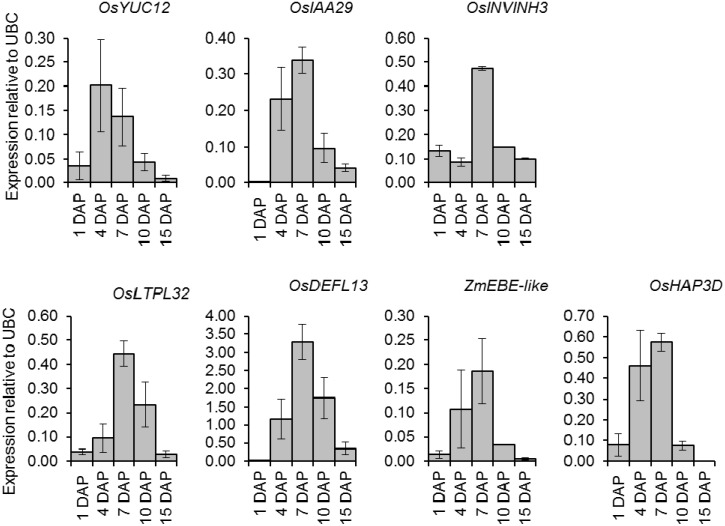
Quantitative RT–PCR results for *OsIAA12*, *OsIAA29*, *OsINVINH3*, *OsLTPL32*, *OsDEFL13*, *ZmEBE-like* *Os11g18140*, and *OsHAP3D*. Total RNA was extracted from developing rice grains 1–15 DAP using a Bioline ISOLATE Plant RNA Mini Kit. Each tube contained 50 ng RNA per 20 μL reaction and a final primer concentration of 0.5 μM. Amplification was carried out using a Bioline SensiFAST™SYBR No-ROX RT-PCR kit in a Rotor-Gene Q (QIAGEN) thermocycler fitted with a 100-tube rotor for 45 cycles. Controls (no reverse transcriptase) were included for *OsINVINH3* and *OsHAP3D.* Expression was calculated relative to reference gene UBC using ROTORGENE software (QIAGEN). Melt curve analysis confirmed that a single product was obtained for each gene amplified. Results represent the means from three biological replicates ± standard error of the means.

To confirm the expression profile of bait genes *OsYUC12, OsIAA29*, and *INVINH3* along with coexpressed genes *LTPL32*, *DEFL13*, *ZmEBE-like Os11g18140*, and *OsHAP3D* in plants of the same variety and grown under the same conditions as our previously published IAA measurements, we carried out quantitative RT-PCR analysis. *LTPL32*, *DEFL13*, *ZmEBE-like Os11g18140* were chosen as representatives from the LTPL, DEFL, and ZmEBE-like gene families, as these genes contained introns allowing for primers to be designed across exon boundaries. Expression data are presented in Figure 4. All genes showed a short-lived increase in expression during early grain development; under our plant growth conditions an increase in expression was seen at 4 DAP (apart from *OsINVINH3*) with highest expression apparent in 7 DAP samples. Large error bars for some 4 DAP samples resulted from one of the biological replicates in which the major increase in expression had occurred earlier than the other two samples. The early increase in expression was seen in most genes when using the same RNA template.

### 2.7. Invertase Activity in Developing Rice Grains

**Figure 5:**
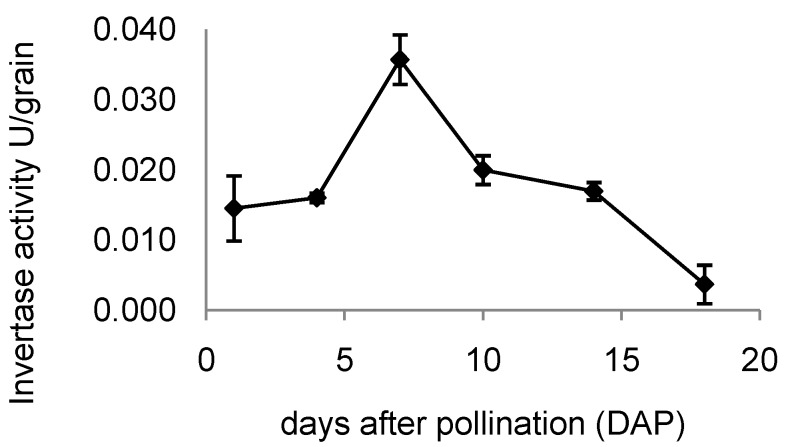
Changes in activity of cell wall invertase expressed per grain extracted from developing rice grains. Values represent the mean of three biological replicates ± standard error.

Cell wall invertase activity during grain development is shown in Figure 5. Total cell wall invertase shown on a per grain basis increased to a maximum at 7 DAP; when calculated on a per g fresh weight basis, no significant difference was seen between samples from 1–7 DAP with a substantial decrease at 10 DAP. 

## 3. Discussion

### 3.1. Expression of IAA Related Genes, OsYUC12 and OsIAA29 Coincides with Endosperm Cellularisation

Previous research using maize and rice has suggested a key role for IAA and CWIN during early grain-fill however the exact roles of IAA and CWIN remain to be established. Our results indicated that the relationship between IAA and CWIN is complex, with differences in expression of three IAA biosynthesis genes *OsYUC9*, *OsYUC11*, and *OsYUC12* during grain development. In this paper we explore the role of one IAA biosynthesis gene, *OsYUC12*. We chose to investigate this gene as to our knowledge there have been no other studies of it or its orthologs. We have shown that *OsYUC12* has conserved orthologs in maize and sorghum, it is specifically expressed in endosperm for a short time during early grain development and this expression pattern also appears in maize. We, therefore, suggest that this gene has a specific and conserved signaling role during grain development. The aim of this work was to identify other possible components of the signaling network that could be investigated. 

An examination of on-line accessible microarray data indicated that AUX/IAA gene *OsIAA29* has a very similar expression pattern to *OsYUC12*; this was confirmed by qRT-PCR. Our quantitative expression data confirm that expression of *OsYUC12* and *OsIAA29* is limited to a short period during early grain-fill. The increase in expression coincided with our previously reported large increase in the IAA content of developing grains at 7 DAP in plants grown under the same conditions [6]. Examination of material under the light microscope indicated that, at 4 DAP, the endosperm syncytium contained many nuclei and a few small irregularly shaped cells. At day seven there were clearly defined endosperm cells containing amyloplasts, as well as differentiated outer cellular layers. The coexpression of *OsYUC12* and *OsIAA29* in the endosperm over a very limited period during grain development, suggests a specific role for IAA during this period. However, OsIAA29 is an unusual AUX/IAA protein that lacks domains I and II [40]. As domain II is responsible for interacting with the F-box protein, IAA co-receptor [41] it is unclear how OsIAA29 may interact with IAA. Dimerization domains III and IV, which mediate interactions between AUX/IAA proteins and ARFs are still present in OsIAA29 [40]. In work with seedling tissue, Jain *et al.* [40] reported very low expression of *OsIAA29* and no expression of its tandem repeat *OsIAA28*. Our observations showing high expression at a very specific stage of development suggest that *OsIAA29* may in fact play a key but as yet unidentified role. This is further supported by the existence of *OsIAA29* orthologs in both sorghum and maize and our recent unpublished observation that the sorghum ortholog is also expressed during grain-fill.

### 3.2. Expression of OsINVINH3 Coincides with Maximum Extracellular Invertase Activity

LeClere *et al.* [42] have provided evidence that *ZmYUC1* expression may be regulated by glucose levels produced as a result of CWIN activity. We therefore investigated the expression of *GIF1* during grain development. Examination of microarray data indicated *GIF1* expression appeared to change little from prior to pollination until 20 DAP. However, we were surprised to find that expression of Os04g49720 (*OsINVINH3*), a co-ortholog of ZM-INVINH1 [8] changed dramatically during early grain-development. Invertase inhibitor ZM-INVINH1 has been reported to bind CWIN during grain development in maize [8]; both ZM-INVINH1 and OsINVINH3 have predicted signal peptides. Quantitative expression analysis confirmed that Os04g49720 showed a large, short-lived increase in expression during early grain development. In plants grown under our conditions maximum expression was found in 7-DAP samples, also coinciding with the peak in *OsYUC12* and *OsIAA29* expression and rapidly increasing IAA levels [6]. We were surprised to also observe maximum invertase activity on a per grain basis at 7 DAP. The unexpected result was confirmed by repetition of the experiment by a second person in the laboratory. Clearly, further work is required to clarify the relationship between CWIN and OsINVINH3, however, our data support the suggestion by Wang *et al.* [5] that CWIN in developing grains may be tightly regulated.

### 3.3. Rice Has 54 INVINH Homologs Several of Which Are Expressed Specifically in Developing Endosperm

Invertase inhibitors have similar structure and are homologous to pectin methylesterase inhibitors [19]. A comprehensive search for members of this family revealed 54 diverse homologous proteins in rice. Sorghum homologs were also included to distinguish between species-specific proteins and those that have been conserved. Although the high sequence diversity resulted in some ambiguous relationships, both sorghum and rice have clear orthologs of ZM-INVINH1. In addition, a small well-supported clade containing the experimentally characterized wheat PMEI suggested that ZM-INVINH3, Sb03g012790, Os01g20970 and Os05g29740 are likely to be PMEIs. *OsINVINH3* is not the only member from the family to be expressed exclusively during grain development. Os02g46290, Os03g43820, Os08g42890, Os08g04670, Os08g04710, and Os08g04740 are also grain specific and expressed maximally at 3–4 DAP. Exploration of data from OS8 [21], OS16 [27], and OS89 [29] indicated that expression is restricted to the endosperm. The seven endosperm-specific proteins are found in four clades; Os03g43820 is present in a large clade quite separate from the others, but including the barley protein F2DCS9 HORVD. Os08g04670, Os08g04710, and Os08g04740 are part of a cluster of nine genes found in tandem; Os08g42890 appears to be part of the same clade though this is not strongly supported. Os02g46290 is a possible ortholog of C/VIF1, an experimentally characterized vacuolar invertase inhibitor from Arabidopsis. The high diversity of these proteins in separate clades of a tree that is known to contain proteins of different biological functions raises the possibility that not all are invertase inhibitors or PMEIs. We suggest that some members of the INVINH/PMEI protein family may regulate other carbohydrate metabolizing enzymes. An investigation of the predicted sub-cellular locations of the gene products, using CELLO [43] and TargetP [44], suggests that all proteins have a signal peptide, however an examination of extracellular and vacuolar invertase inhibitors from potato [17] shows that domain prediction programs cannot reliably distinguish between these two sub-cellular destinations. 

### 3.4. Putative Signaling Proteins Coexpressed with OsYUC12, OsIAA29 and OsINVINH3

The observation that *OsYUC12*, *OsIAA29*, and *OsINVINH3* are expressed exclusively in endosperm samples from developing grains 3–10 DAP suggest they may be co-regulated and may be part of a common signaling pathway. A meta-analysis of microarray data indicated genes from three families, *CRPs*, *ZmEBE-like*, and *INVINH/PMEI-like*, all encoding putative extracellular proteins, are prominent among genes coexpressed with *OsYUC12*, *OsIAA29*, and *OsINVINH1*. The CRPs come from two distinct groups; the so-called lipid transfer protein-like group and the defensin/defensin-like group. We note that despite their reported antimicrobial activity, defensins have recently been shown to have a signaling role earlier in maize reproductive development [45]. In addition, the CRP MEG1 is essential for the differentiation of ETCs in maize [46]. *OsLTPL29/OsPR602* and *DEFL OsPR9a*, as well as *ZmEBE1*, *ZmEBE2*, and *ZM-INVINH1*, are also expressed primarily in ETCs and ESR [38,47]. Differentiating transfer cells in maize kernels accumulate high levels of auxin transporter *OsPIN1* transcripts [4]; the BETL and ESR also show high levels of PIN proteins as well as an accumulation of IAA itself. The specific localization of rice proteins found in Table 2 should be investigated to determine whether they are also restricted to the ETCs and/or ESR. 

Genes with a mutual co-expression rank of <20 included also *OsHAP3D* [34] and Os05g07850, a putative leucine-rich repeat receptor-like kinase. *OsHAP3D*, also referred to as *OsLEC1a*, encodes a nuclear factor YC protein, homologous to Arabidopsis LEC1/L1L. These Arabidopsis proteins have a crucial role in embryogenesis [48] including the up-regulation of *YUCCA10* [49]. Rice has two homologs of LEC1, OsHAP3D, and OsHAP3E. An examination of their expression profile via PLEXdb indicates that both are expressed in developing grains with a peak at 3–4 DAP. However, whereas *OsHAP3D* is expressed in endosperm, *OsHAP3E* expression appears to be restricted to the embryo. Os05g07850 encodes a leucine-rich repeat receptor-like kinase which has a predicted trans-membrane domain and serine/threonine kinase domain. It is homologous but not orthologous to the brassinolide receptor BRI1 and aligns in the same clade as Arabidopsis NSP interacting kinases. A partially homologous receptor-like kinase has been shown to form part of a signaling network that also includes a DEFL during fertilization in maize [50]. The identification of these coexpressed genes provides a framework for investigating protein-protein interactions that could form part of a signaling network.

## 4. Experimental

### 4.1. Bioinformatic Analysis

Rice protein sequences were downloaded via Phytozome 8.0 [51] from MSU Release 7.0 of the Rice Genome Annotation; protein sequences for ZM-INVINH1, ZM-INVINH2, ZM-INVINH3, and experimentally characterized pectin methyl esterase inhibitors (PMEI) and invertase inhibitors (INVINH) from wheat, potato, tomato, and Arabidopsis were downloaded from Uniprot. The phylogenetic analysis of protein sequences was carried out in MEGA5.2 [25] using the Neighbor-Joining method [26]. MUSCLE [27] was used for multiple sequence alignment. The bootstrap consensus tree was inferred from 500 replicates [28]. Evolutionary distances were computed using the Poisson correction method [24]. All ambiguous positions were removed for each sequence pair. Normalized microarray data from accessions OS5 [20], OS8 [21], OS16 [22], OS17 [5], OS44 [23], and OS89 [24] was downloaded via PLEXdb [14] and analyzed in Excel. Pairwise mutual coexpression ranks were calculated as per Obayashi *et al*. [33].

### 4.2. Plant Material

Rice grains (ssp. Japonica cv*.* Jarrah) were direct sown into flooded soil-filled cylindrical plastic pots and thinned out to three plants per pot when the seedlings had reached the 2–3 leaf growth stage. Plants were grown in a glasshouse under a natural light with day/night temperatures 28 °C/18 °C. Plants were fertilized fortnightly using Aquasol^®^ (8 g/5 L). When spikelets in the top half of the panicle had reached anthesis, panicles were tagged to record the date; panicles were harvested at 1, 4, 7, 10, and 15 days after pollination (DAP). Grain samples were placed in microfuge tubes, the weight and number of grains recorded before freezing in liquid nitrogen and storage at −80 °C. 

### 4.3. Quantitative Reverse Transcriptase PCR

Total RNA was extracted from 60–80 mg grain samples following instructions for the Bioline ISOLATE Plant RNA Mini Kit. RNA concentration and 260/280 ratio were determined using a NanoDrop ND-1000 Spectrophotometer (NanoDrop Technologies, Inc., Wilmington, DE, USA). Only samples with A_260_/A_280_ of 1.8–2.0 were used for further analysis. The RNA quality was also checked using agarose gel electrophoresis for two clear bands of 18s and 28s rRNA [52]. 

Gene-specific primers (See Supplemental Table S2) were designed with melting points in the range 59–61 °C and product sizes between 100–150 bp. Primers were tested for amplification of a single product of the expected size using a QIAGEN OneStep RT-PCR kit (QIAGEN, Limburg, The Netherlands), with agarose gel analysis of products. To avoid the possibility of amplifying genomic DNA, either left or right primers were designed to span an exon-exon boundary. Controls (no reverse transcriptase) were included for *INVINH1* and *OsHAP3D* genes, which had no introns; amplification was not detected in the controls. Quantitative RT-PCR was carried out following manufacturer’s instructions using 50 ng RNA per 20 μL reaction, a final primer concentration of 0.5 μM and reagents from a Bioline SensiFAST™SYBR No-ROX RT-PCR kit (Bioline, London, UK). Reaction tubes were prepared by using a QIAgility robotic liquid handling system (QIAGEN). Reactions were monitored in a Rotor-Gene Q (QIAGEN) thermocycler fitted with a 100-tube gene disc rotor. The amplification program included 48 °C min, 95 °C 2 min and 45 cycles of 95 °C 5 s, 58 °C 10 s, and 72 °C 5 s. Expression was calculated relative to reference gene for ubiquitin-conjugating enzyme E2 (*OsUBC*) [53] using ROTORGENE software (QIAGEN). Melt curve analysis confirmed that a single product was obtained for each gene amplified.

### 4.4. Invertase Assay

Rice grain samples were extracted using a procedure modified from Tomlinson *et al*. [54]. Samples (200 mg) were ground to a fine powder in liquid N_2_ in a mortar and pestle then transferred to a close fitting glass homogenizer and ground further with 1 mL of extraction buffer (pH 4.8, 100 mM acetic acetate buffer, containing 8 mM MgCl_2_, 2 mM EDTA and 12.5% (w/v) glycerol, 10 mM dithiothreitol and 1 mM phenylmethanesulfonyl fluoride). The homogenate plus a 400 μL wash were transferred to a 2 mL microfuge tube and centrifuged at 16,000 ×*g* for 10 min. The pellet was washed and re-centrifuged twice with 500 μL of extraction buffer before resuspending in two volumes of salt extraction buffer (pH 4.8 100 mM acetate buffer, 1 M NaCl). Samples were extracted for 1 h on ice with gentle agitation, as well as 3× sonication for 60 s. Following salt extraction, samples were centrifuged at 16,000 ×*g* for 10 min and the supernatant retained for assay of CWIN activity.

Reaction mixtures containing 80 μL of enzyme preparation, 10 mM sucrose and pH 4.8, 100 mM acetate buffer to a total volume of 440 μL were incubated at 37 °C for 30 min with gentle shaking. Three 40 μL aliquots were taken for glucose determination at 0 and 30 min. Glucose in the reaction mixture was measured using the Somogyi-Nelson method [55]; and calculated in comparison with a set of standards containing 5 to 100 μg glucose.

## 5. Conclusions

In conclusion, we have shown that the endosperm-specific IAA synthesis gene *OsYUC12* and the AUX/IAA gene *OsIAA29* are coexpressed, with expression limited to a short period during endosperm cellularisation. Extractable CWIN activity, as well as expression of putative invertase inhibitor *OsINVINH3*, also reached a maximum at 7 DAP, coinciding with maximal expression of *OsYUC12* and *OsIAA29.* The possible regulation of CWIN activity by OsINVINH3 needs to be considered as part of any investigation of the role of CWIN during grain development. We have shown that rice has at least 54 genes encoding diverse proteins homologous to invertase inhibitors and PMEIs. In addition to *OsINVINH3*, a rice ortholog of *ZM-INVINH1*, six other *INVINH/PMEI* genes from widely divergent clades were specifically expressed in endosperm during early grain-fill. We explored other genes co-expressed with *OsYUC12*, *OsIAA29*, and *OsINVINH3*. Seven out of the nine genes with MR<20 appear to encode extracellular proteins homologous to molecules expressed exclusively in ETCs/ESR of other plants; these include six cysteine-rich proteins, some with similarity to BETL1 from maize. In addition, the presence of an endosperm-specific *LEC1* ortholog, *OsHADP3D* and a receptor–like kinase gene suggests that these genes and their encoded proteins are a priority for experimental investigation in regard to a signaling network regulating differentiation of ETCs in rice. The expression of *OsYUC12* is distinct from *OsYUC9* and *OsYUC11*. It has orthologs in sorghum and maize; the maize ortholog is also expressed for a short time during grain development, suggesting conserved subfunctionalization. Expression of *OsYUC9* but not *OsYUC12* is reduced in the *gif1* CWIN mutant. We suggest that further investigation of the specific roles of three IAA biosynthesis genes will be necessary to reveal the full extent of the relationship between CWIN and IAA.

## Supplementary Files

Supplementary File 1
