# Supplementary material for: Auxin and Cell Wall Invertase Related Signaling during Rice Grain Development
Source: Plants (Basel). 2014 Feb 7;3(1):95–112. doi: 10.3390/plants3010095 (PMC4844310; doi:10.3390/plants3010095)
Supplement: Supplementary File 1 [file plants-03-00095-s001.pdf]

**Supplemenary Material**

**Figure S1.** Phylogenetic tree showing relationships between homologues of *OsYUC9*, *OsYUC11* and *OsYUC12* from rice, sorghum and maize. The tree is rooted using *OsYUC8*. The tree was produced in MEGA5.2 [25] using the Neighbour-Joining method [26]. MUSCLE [27] was used for multiple sequence alignment. The bootstrap consensus tree was inferred from 500 replicates [28]. Evolutionary distances were computed using the Poisson correction method [29]. All ambiguous positions were removed for each sequence pair. Highlighted sequences are encoded by genes expressed in endosperm during early caryopsis development.

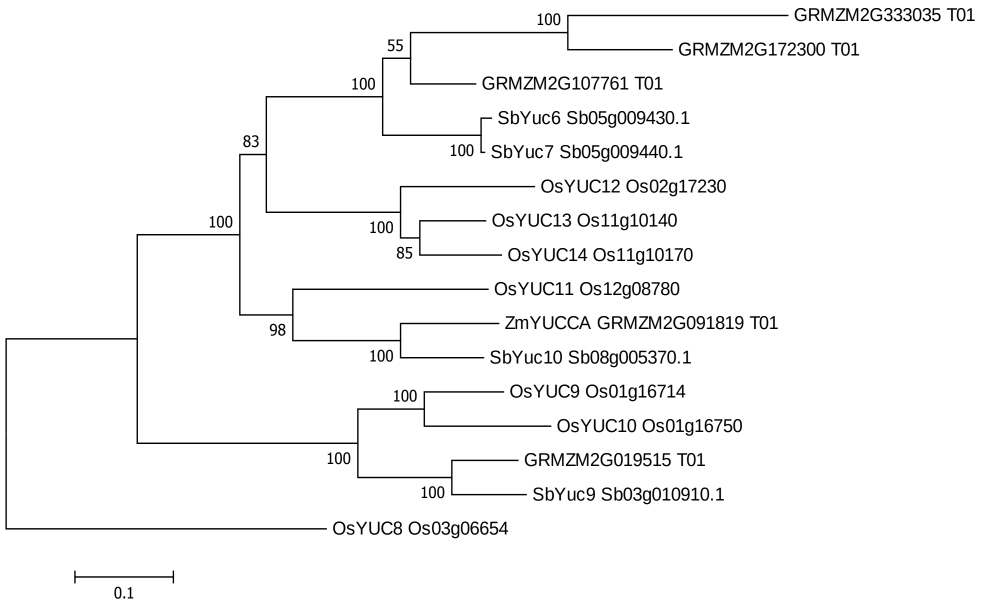


**Figure S2.** Expression of maize orthologue of *OsYUC12, (GRMZM2G107761_T01)* during grain development using RMA normalized data from Genome-wide atlas of transcription during maize development [13] accessed as accession ZM37 from PLEXdb.

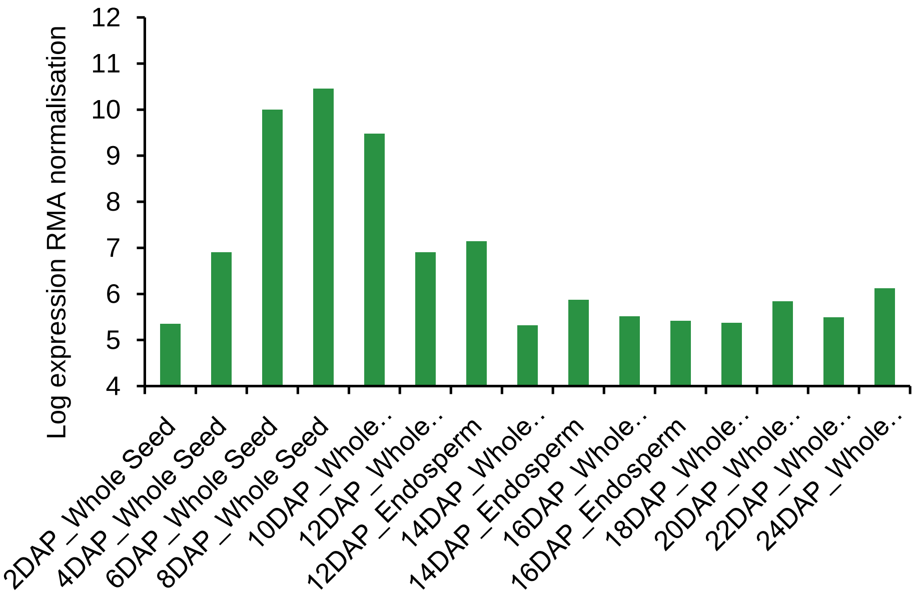

**Figure S3.** Multiple sequence alignment of INVINH/PMEI protein sequences used for the phylogenetic tree in Figure 2. The alignment was carried out using MUSCLE [22]. CLUSTAL multiple sequence alignment by MUSCLE (version 3.8) The sequences shown below as **A**, **B** and **C** are contiguous.

```
Os08g04760      ------------------------MVKPIGSLAIAAVVPLLA-------------------------------------ALCLVSCTGGDARWCAPVPSMTVEQACDAVC---GTAHMLK
Os08g04710      ------------------------MNTIPLPLRLLVAVSL-------------------------------------AVAGVAGDDEKTCPGAPTM----TVESACRNVS---GTQAMYD
Os08g04740      ------------------------MNTIPFLSPLLVAVSLI------------------------------------VAGAGAGDETTTCPGAPSM----TVESACRNVS---HTQAMYD
Os08g04670      ------------------------MEKTTFAVLSLSLLLLAG----------------------------------------GGGADACEDYDV------PMMSAAVAFERASTGRAMSE
Os08g04700      ------------------------MEKPAAVFAVLSMSATAT-----------------------------------LLLLLAGGGGVADACSDWDVPSMSAAAVCQRAS---TGRTISQ
Os08g04650      ------------------------MERSATVFVALTLLVAGS-----------------------------------VVVVVVVDACDGVPRM-------SAVEACKQAS---VGPAMSR
Os08g04660      ---------------------------------------------------------------------------------------------------------------------MRQ
Os08g04690      ------------------------MSVALTLSLLLTAASLLA-----------------------------------GVADGCDGVPRM-----------SAVDACKQAS---TAGVMWQ
Os08g04640      ------------------------MTRASATGFLAAVAMALA-----------------------------------VAAVVVDGCDNVPSM--------SMDDACLKAS---TSQPLLA
Os08g04680      ------------------------MNKRADAIVFLLAMAVVV-----------------------------------IVVDACDGVP-----------SMSLEDTCQKAFG--TAAAPTD
Os04g36770      ------------------------MAARLVFSLLLTVACSHA-----------------------------------ALAAAASSS--------------AVEDTCAKATASGSRKDLAP
Os08g25070      -----------------MTTAMASSSSRGVTVPFLMILPLLF-----------------------------------VVAALPAEVTVTGDG--------ILLPSCKTVGGG-STYFDVQ
Os12g03510      ------------------------MVSSHGIVNAFFLFAVAL-----------------------------------VAASQAQHAANADS---------FMSGACKIVAGSSSGVISVT
Os11g03740      ------------------------MSSINGVSILFLLAVLL------------------------------------PASQLAAGIDS------------ALYGACKTVAGN-SGVVSVT
Os11g03750      -----------------------MASSNNGVSALFLLAVLLI-----------------------------------AASQLAAGINS------------AVYGACKTIAG-GSGLIGVT
Os07g14130      ------------------------MADHQTLSAVFFLLLVP------------------------------------HAACMAMAKPSSST---------LLQDKCELYAA--GDRPSYD
Os02g46360      ------------------------MRRLLVPVLLLMLGLSTC-------------------------------------------------------ESSVLQDTCKSVAAG-HKYVTYN
Os04g49730      ------------------------MKLVRSFSPLIVFSLLLL-----------------------------------LTSSTTSRAS-------------LVDDACTSFAAS-HADIGYA
Os04g49720      ------------------------MAFLRALSPLVFTLLLLL-------------------------------------AASTSDAS-------------ILEETCKSLAGN-HTAIDYP
Os05g20570      ------------------------MGPAHGHLVLLVAAVALLSAGF-------------------------------LPHAVAKKGGAVPP---------AVADIC-------SRTPFPD
Os01g14940      ------------------------MARSLAHATLLLVVAVVA-----------------------------------AAACWTTAAGENE----------EVAEICKK-------TSYPE
Os03g61530      ------------------------MAAASLRLVVVAAAVAIS-----------------------------------LRGAAATAV--------------TVEDAC-------RHTRHEA
Os01g20970      ------------------------MASSPYHAAVGVAVLAILL----------------------------------AAPAAEAGAPTAAPLANY-----SLEDACK------KTGPHYG
Os05g29740      ------------------------MAASSSVLVVVAACLAAA-----------------------------------LVCLAANVAPASCARATAALPHASIAETCSF-------VDDHK
Os02g01300      ------------------------MATLCGSVCFLFVIMVIA-----------------------------------SSPAAVAAAGED-----------IVEETCGQCSRS-NPNVNYT
Os03g40900      --------MFLALVILLAVAAGSWQPAATSTPATVSPAPAVS-----------------------------------PVAAPAGELGGGGAGARRDQDREFVRGCC-------ARTLYPR
Os08g12600      ------------------------MATVLVVLMVVSTGGLSPPCAAAAKEEKPVVVL--------------------PPAAAPGEAPFAEAAA-------FVRSCCETALP--AERDASS
Os08g12160      ------------------------MSRSSSSMATVLVVLMVVSAGGLSPPCAAAAKEEKPVVVL-------------PPAAAPGEAPSADAAA-------FVRSCCDTALQ--ADRDGSS
Os08g12230      ------------------------MSRSSSSMATVLVVLMVVSAGGLSPPCAAAAKEEKPVVVL-------------PPAAAPGEAPSADAAA-------FVRSCCDTALQ--ADRDGSS
Os08g12520      ------------------------MSRSSMAAVFLVVLIAINVVCLSPQGATAAPEGELHKLNLEL-----------PPPLPPGKEPSTEAYR-------FTQQVCDFGTSTFTEQMIGA
Os08g19780      ------------------------M----------------------------------------------------TAVLLQEPSPAAYQ---------FSRTICRIP----KDPIYKI
Os03g43820      -------MDLGQFVSTKVVSSIFTMASSPSLALSFLVLLVLATFTTTTVLATNNL----------------------PPSSAPALSPASSAAAKE-----FLRATCT------SKSELPE
Os10g10700      MEQVHLRSLNFTPNPIVVLQPQNQTQPPYNSLLNLSLKPLCISTAKPATASSSRNNGASSSHDRRLLRLRRRTTVVGFSGDSQGGHPAAPPCVAPPAAVAFLRASC-------ASTLYRL
Os10g10630      ---------------------MPSSRAGPRHVVLVMLFFAAAV----------------------------------AARGADAASPVTTRPCGAAAAASFLCSRC-------ATTVYPA
Os10g10560      ------------------------MAAASATQATAAEAAA-------------------------------------PCSTTKTESTSSSS---------FLRARC-------ATTRYPD
Os10g10620      -----------------------MAPLPPRAISLVFLFLFFT-----------------------------------AASATRRLDDAGGQPTAASSATAFLRSRC-------ATTRYPD
Os10g36500      ------------------------MARSLSLLLLLVACFLVAGATAAR-----------------------------PAPSTSGAAAISS----------FVRSWC-------AGTKYPA
Os04g01570      ------------------------MAITRRHPPLLLLFLAVAAA---------------------------------ATIAGASQSTRPRPAVSPAAAADFVRRSC-------RATRYPQ
Os06g49760      ------------------------MSRSSLAMALLLAAFAA------------------------------------AATANSGAAPAASD---------FIRKSC-------RATQYPA
Os08g24160      -------------------------------MALLLLAFFAA-----------------------------------AATANGGAAPAASN---------FIRKLC-------RATQYPA
Os12g37480      ------------------------MASSPPLVACVLLAAVVFTAVAPPPAGAVCVPRNGKAAPGKPGMSPAPPKK--LTPAPPTTPPPKAKPILPGPGGDLVKALC-------AKTDYPV
Os08g01670      ------------------------MASSMAPAAAMAILLLALLMPATLCSRSGPPSSKHGH----------------GGHAKRAPPPASPVVPVAPQAAALVRATC-------NSTAYYD
Os12g18560      -----------------------MASMAPSAMVLIVLLVLVVLPSSTLCSRAGPSSKHGHGGG--------------HAKRVPPPASVPPPPPPPPAPAALVRATC-------NSTSYYD
Os02g46290      -----------------------------MHPIIATLLLAAA-----------------------------------IAPGLTVGSPS------------LINETC-------TATSNYG
Os02g01310      -------------MSIRFDPMESRSSSSSRSVVMMCCVLMLM-----------------------------------IAAAAHAGEQEAEECASPM----SIEEACRGASET-HHAVAYD
Os05g05640      ----------------------------MKASSLAAVAAAVL-----------------------------------VAAACSGRAPVAEA---------TIESTCGAAAKG-DRRVDVG
Os03g01020      ------------------------MRMSKALAAVVAISVSLS-------------------------------------AAAMGVDA-------------TVESTCSDAAAS-DKRVHLA
Os02g33130      ----------------------------MRPSSLALVVIVAT-----------------------------------AILTLSGAAHA------------DVQGTCKAAAGI-DSRISYK
Os02g33380      -----------------------MAQRASRRPAAAAAAVVVA-----------------------------------VVLAVSGGVGA------------TPETACRAAAEE-DRRVDYE
Os02g33390      ------------------------MLMMMKPYTAALLAVAVA-----------------------------------VLGVVAVDA--------------TVVTTCRAAADA-DARVDYG
Os08g42890      ------------------------MARPAAAVTVLLAVVVLV-----------------------------------SVAASLPSAVVGDAR--------FVARTCK--------RTNHT
Os01g02070      -------------------------------------------------------------------------------MADAG----------------FIATTCN--------KTHNA
```

**(A)**

**Figure S3.** *Cont.*

```
Os08g04760    LCLRTLQPRARRGGGEHRRRRDDAVTRYVGAAARGALDAYAATAAA-KRGMQYSA--------ALPAEERTA---HERCM-AGYD-LAVRFMGRVA----GDLA----------------
Os08g04710    TCRDALAGVA--DPLS-----DHDVTVYALAAAHGAAASAEATMGAAIDEVRNNR--------SLPGDERDA---YMECA-VDYS-TALHAMGSVV----DKLD----------------
Os08g04740    ACKGALAGVP--NPMS-----DHDATVYALAAARGAMASADATVVAANERVTYNG--------SLSGEEKGA---YEGCV-EAYA-AAEHAMGAVL----NKLG----------------
Os08g04670    MCADEVGT----ATAP-----DQEVTDFVFAAVSAAARSCGATARA-VRDMARDP--------STPPGAREA---GRACD-GRYG-EAMARLADAV----GHLN----------------
Os08g04700    ICADEGGT----ATAP-----EQEVTVFVLAAVNAAARSYEATVGSYLWPLATDP--------SAPGAARAA---AQACV-HKYR-DARAAADGRRGGPPERLR----------------
Os08g04650    TCAETLG-----TSAD-----EQGATDFMVAAANAATESYKAGKEA-VGKVLSNP--------LAPDGERLP---CLVCA-NKYD-DASMLVASTA----DDAK----------------
Os08g04660    LCAAMLG-----TSPE-----PKEVTGYVFAAMDSNIAQFLISYEA-ADKVRQDA--------ASSEQLSAA---CETCM-GKYD-LAKMKMVYSG----NRLR----------------
Os08g04690    LCVRELG-----ASPE-----PEEVTGFVAAAMRANREAYGVSYDA-AEKVRVDP--------SSPAGLATV---SGYCE-GKYD-TAQELMTGWI----DRLP----------------
Os08g04640    LCHGELL-----NAPE-----SGEVTVYAVISARWAQWAYEATSRA-AAGLLGNNNA------SLAAGERAA---YEACV-ARYA-SAKARVMAVQ----TQLL----------------
Os08g04680    ACGAPPCITP--MHVY-----CVSATVFAVAATKYAKESYESTMEAAFRALQNA---------SLPGDERAA---CAACRDTYYA-QARSSTVAAM----NLLA----------------
Os04g36770    FCVSTLQAA---PGSA-----GADARGLAVIATNLTLANYTAAYAT-IKALQRRG--------GWSERERAA---LATCR-QLYI-EALNVVHSAI----HALN----------------
Os08g25070    FCLDALGSVG--AGAG-----ARSYRDLAAVAVGLLTANATSTSVK-IDALLRGGGGGG----KVDAATARC---LRSCR-ALYA-GIARRQPGCA----AAVR----------------
Os12g03510    FCMDALGSDS--RSLS-----ASYYSDLAIIAIDLLTSNTTSTKAK-IDNILKDDGNGL----KPGDATTVC---FQSCQ-AAYA-SVLQGQLGIF----YNVQ----------------
Os11g03740    FCIYALSSDN--RSHD-----AAGFKDYAVVTVDLITANATSTKSK-IDGILQNGGGG-----GAGDAKRRC---LQSCQ-AAYA-GVLRAQPGIV----ADVQ----------------
Os11g03750    FCIDALSSDN--RSSN-----VGSYKEFAVIAVDLLTANATSTKSE-IDAMLRSSG-------GGGDATTRC---LKSCQ-AVYG-GILQVQAAVG----AAVK----------------
Os07g14130    YCIRTLRAD---RASA-----TDDERGLAAIAARIARATAVATGAK-IARLQRG---------ETAPARRDG---LAACA-AEYA-AAVRRLGRAA----RDVV---------------S
Os02g46360    DCIKAFQADS--ASAT-----AADARGLAAIAARIAEKAANATSAR-IAALRAA---------EKDARRKDR---LGVCA-EVYS-DAVDQLGETA----EDIA----------------
Os04g49730    YCVRFFQSD---EGSA-----TADRYGLAAIAVKISAATARGTAKR-IADLQDL---------ERDKRRKDC---LSACG-EVYD-SAVDSLDEAA----KGIA----------------
Os04g49720    YCVRFFQAS---KESA-----TADTHGLAAIAVRITEAAAEGTAKR-IAALRSS---------HKEEKMQEC---LRISS-DLYA-YMLAVLGNEA----KAAA----------------
Os05g20570    TCKSTAGRHA--SKYP-----VIDVVAVLNMQVDAFSKRTAHARER-VTKVSR----------GAQPQQTQA---LSFCD-TMYM-NTQDTIGAAE----RAIT----------------
Os01g14940    VCIATAGKQ---ASKY-----GAAVDPLAVLNMQVDAFALRTEAAR--KHLTEAVK-------TATPKAAKA---LDLCD-SLYL-DVEDNLGAAR----RAIG----------------
Os03g61530    YCVKALSARPE-SRAA-----ALDMPALAEAALSMAAESGAAATSF-VRNLAK----------MPGGMPPEC---LEGCV-AKFQ-EAVAELRRSE----AAME----------------
Os01g20970    LCIVTLSAD---RSAK-----SSDTVGLARVAVLAAQKNASETATY-LSSIYDDDSIE-----KKTVQLQQC---LEDCS-ERYE-AAVEQLTDAT----VALD----------------
Os05g29740    LCEESLSSLPL-TARA-----AADARVLARAAVLLARQNATATAAY-LSHLHAAAAAAAD---DGDDADHRC---VGDCT-VRYD-RAVAYLGDAA----AALD----------------
Os02g01300    LCVSSLSGYP-----------GADLHGLALFSTMPLRSALATIASE-ARVLRDRA--------PPGSPPRSC---LDSCL-EVFR-DAAYNLGNAV----AAIE----------------
Os03g40900    LCTAALSPY---AAAV-----GSSHARLAVPSANLTAGTINSLGGR-IPSPSTT---------GTTESPAGA---LGDCA-EAVA-SAADLAARAA----GRLD----------------
Os08g12600    FCYDELLPY---ADSF-----GGNQVKVTEVAATILSNNLSAYLDE-LRKVQ-----------DGAGKGDQN---LMACV-DGFTDATNVNITKEALDSLGRLA---------------A
Os08g12160    FCYYHLLPY---AAFF-----EGNQVKVAEVAATILSTNLWVYVDQ-LRKVQ-----------GGAGKGDPN---LNACV-DDFSVAAGENITREALQSLGRLA---------------A
Os08g12230    FCYDHLLPY---AAFF-----EGNQVKVAEVAATILSTNLW----------------------------------VYACSLATY-----------------------------------
Os08g12520    RCYDVLIPY---GESF-----KGNQVKATQVATIILVTKIQALTSE-AYAI------------RDMGIRDEN---INNCG-SFF--AIGETFSDTTNSTLYALE------------RLTA
Os08g19780    CCFGELLPY---AESF-----QDNQMKATQVATIILLTKIQALDRE-----------------------------VGAST-NSVL-AALDRLAAAG-----------------------
Os03g43820    LCFDILLPY---ASSF-----NGSQGKVARASAAIAIERHRGLLDE-LRGLKPGPG-------DVGAERRMLVMLLSDCV-RDFD-ATYMFADETL----ARID-----------FLVSG
Os10g10700    TCYDALIPS---GCAF-----QTSTVKLARAAADVNAASLKNLTAR-AKELVAHGVP------GEAPAIAAE---IRDCASASSSASGHAKETAAELARLDAMG-------------DAA
Os10g10630    VCYDSLLPY---AGAF-----QDSRVRLARAAADVAAARLRDFSAS-LDELVHGSGDVGAV--TTPPRVAAA---VRDCV-GTVS-SAAGLARRSS----AALD-----RLDAGAAAGGG
Os10g10560    VCYDSLLPY---ASTF-----QTSHVKLAVAAADVAAAHLRAFSARVKDMLLHRGGGSEEAAASSGGGAARVDAVLHDCA-STIS-AAANLAKRSS----AELTRLDADTAASTETSTSG
Os10g10620    VCYDSLLPY---ASTF-----QTSHVKLAVAAANVAAAKLRAFSAR-INDLLAQ---------GGAARVDAA---LKDCK-STIS-DAGDLARQSS----AELG-----QLDAGAAAAGV
Os10g36500    LCDATLAPY---SAAV-----GASPAKLAWAALTVTLSAARNATSA-VRSIAARSSSS-----HLPPVAAEA---AGDCA-SELG-DGVDALRRCV----DAMAR----VAVGEESSSTA
Os04g01570    VCERSLMPQ---APAV-----GRSPRLLAQAALTVGADRARSCSGY-LGGGGSSSSSKR----SGGGGRGGA---VGDCA-DTLR-DAEERLRQSA----AEMS-----------RMGRS
Os06g49760    VCVQSLASYGGAGAPP-----PRSPRELARAALSVSVDKARSASTY-VGHICGPGGG------RGGAGGAGP---VRDCL-ENMA-DSVGHLRDAAQELGGNMG--------------RA
Os08g24160    VCVQSQASYG--GAPL-----PRSPPELVHAVLSVSVDKAQSVSMY-VGRICGP---------GGSRGVAGP---VRDCL-ENMA-DSVGHLCDAAQELGGNMG--------------RA
Os12g37480    VCQMTVVPPPAAGAAA-----KLDATAVLRLAMGAVRAKAAAAKKA-AGALAADA--------RTPALAKPV---LRDCM-DSYD-DIAYSLDEAD----KAMA----------------
Os08g01670    VCVSALAAD---PSST-----TADVRGLSAIAVSVAAANASGAAQA-AAALANGTAPLA----AAAAGDGTVQALLRACA-GKYG-DARDALAAAK----ESMG----------------
Os12g18560    LCVAELSAD---PSSA-----TADVRGLSSIAVSAAAANASGAAQA-ASALANATDAGTTAGVAGDGGGAVVQRLLATCA-AKYG-DARDALAAAK----GSIA----------------
Os02g46290    YCLGVLSADP--AGAS-----ATDKRGLAIAAANITMRNVTSTVRV-LTELVEE---------------------LKLCI-KYYQ-DMDDLVASAI----DDLR----------------
Os02g01310    HCVASLTADPRSSSSE-----ARSLHGLAMLATRMAIDHATSTVCK-MDDLAELEPEDS----SSPSDARAR---FEHCL-EQYG-GAADLLRDAL----DNLK----------------
Os05g05640    FCARQFASY---HGAA-----EADAWGLAKIAALIGVNLADDAVFD-IGAGKIRPSP------GGGAKGDKA---MDACA-KAYD-AVGVAFAEAA----DELG----------------
Os03g01020    MCLSQLGHHR-----------DADAWGLAKAATLVGVDKADLAADD-IKELEAG---------ASTAGIKPA---LAECA-KQYR-GVGFAFASAH----DVIN----------------
Os02g33130    FCVSKLSNH---HLSP-----DADTRGLALIAASLGISNAEDTVFD-IKGLVAKP--------GTGAKAKPL---LARCQ-ELYN-EMSFVFAEGY----DCIN----------------
Os02g33380    FCVSRLSHH---HDSP-----DADTWGLAKVAADVGVCIAGDAAYDAKAKLQA----------AKAGGEREA---LERCA-ELYD-RMGSAFAAAY----DDIN----------------
Os02g33390    FCVAELGKH---HDSP-----GADAWGLAKVAALTGVVDADNAAYD-ARDLLA----------KGGGGAAAA---LARCG-ELYR-AAGFAFAEAH----DDIN----------------
Os08g42890    ECVKMLSADR--RSAR-----ATTVHQLAGIAVDIAAATVKSSAAAVYGKFLE----------NHGQVLELT---LLECW-WMYDLAAGEAQAAVD----AYSS----------------
Os01g02070    KCVAVLTANP--DSAD-----ASTVSDLAGVALDLAVAAASDAGAL-INDRSSRY--------GGGAPEGEA---LRACSGAYFD-AANDLDIDAH----DSLG----------------
```

(**B**)

```
Os08g04760    -----SCETAAARLRDDCD-------GSLA--GMDACRRKLFG-------------YPASPLIHHISCLIRTDLEGVPDKERQLEFYMETTSTTRIVSILISLVLLEQGDTYGYKYKPPR
Os08g04710    -----GC--SFDGLAGDYL-------NGLI--DVENCRDRVLK-------------LPASP-LYAMVLVDR--------NKAGMA-----L-------FLGKLLGI-------------
Os08g04740    -----ACSFGGGLADDYMS--------GLL--AVESCRDRVIK-------------LPASP-LYAMVLVDR--------NKVGLA-----L-------FLGKLLGI-------------
Os08g04670    -----GC--QLVELSADAP-------AAIA--AVDDRTTALLP----VVG------F--SP-LYNRVIGDR--------DRCVLA-----L-------RLISILLHHPGSTLA-------
Os08g04700    -----AG-------------------GARQ--GRSGGRRRRRR-------------------LRHRAAAGV--------RR-----------------ELGVVQDGGR-----------
Os08g04650    -----RC-KLSADSLPDLV-------TAVS--AVDECATKMFE----ESG------NT-TS-VYATAITNR--------DWTVLV-----L-------RLATLVVPRQQLS---------
Os08g04660    -----SC-DMSPVTREDLL-------TAVV--GIDDCATLLLN-----AA------GDKTP-LRRMVLLDR--------DRAVLL-----L-------QLAILLLPNKS-----------
Os08g04690    -----GC-DITADIRVDLA-------SAAA--AVDECATLLLQ-----NG------GEHTT-LYQMVLLDR--------DRAVLA-----V-------RLAILLVPNKV-----------
Os08g04640    -----EC--SYASPKQELI-------DARV--DVEACGGELSR-------------FAASP-LHAMNADDQ--------LKATLA-----Y-------ELTGLIIGK-------------
Os08g04680    -----EC--SLGQLGGEYA-------AAAD--AIKACRDAQSK-------------LQ-SPAIYGLAVSDL--------MVAALASGLGELVICQAVTWLINLLQEHEAQWPSRQRGESG
Os04g36770    -----TG--QTQAYVADMG-------VVRR--AATGCEDAFGFGGGGGGGGVGNQLATESP-LHKVDDDAI--------NLTTVA-------------TLIVLIL---------------
Os08g25070    -----GG--RLGEARSSLE-------ESAA--AARRCEDGFRG-----GN------AT-SP-VTAEDDAAF--------KLAKLG-----V-------ALLGFA---------------
Os12g03510    -----AG--RFPEAMSALE-------KAAN--MVEECEKGFGK-----SN------VK-SL-LTTENHDSF--------ELA-----------------KLGALLLNEEH-----------
Os11g03740    -----GG--RLPEAISALE-------KSAS---GAGMREWFRQ---------------------------EQR------------------------DVTGHRG---------------
Os11g03750    -----GG--RFQEAISSLE-------KSAS--AVKECRDGFGK-----SN------VT-SP-LSVENDDTF--------QLA-----------------ELIVLLIRDEP-----------
Os07g14130    RSRGGAGAREMREAQTLLA-------EVTG--APERCDVAFEA-----AG------GQGSP-LDAVDRDRA--------RRRGRV-------------GLRHPAADEADVM---------
Os02g46360    ----RGGDEATQDAVTQLS-------AALD--APGTCEDAFGE-----AD------DA-SP-LAPEDAEFA--------KLATIA-----L-------AVAASLSPPPSTPATMD-----
Os04g49730    ----SRSADGLRDAVTVLS-------AALD--TPDTCEDGFRE-----LG------LA-SP-LVAEDEEFS--------KESAIA-----L-------GVTSALSPPS-----------
Os04g49720    --LVDGG--AQHAAASPPT-------PDVARYVADGCEGRFRG-----NK------ET-LP-LVAEYAEFR--------QSASIA-----L-------ALIEAISPPSDQ----------
Os05g20570    -----FR--DKATAKIMLQ-------LAVQ--DFESCDRPFKQ-----SG------IN-NP-LEKIDVELN--------QMAQNC-----M-------TLASMI---------------
Os01g14940    -----FK--DAVTIRAMMG-------MAAQ--DMQGCDEQFRK-----VG------EK-NP-MDHFNRSLL--------EMSEIC-----R-------SLSNMI---------------
Os03g61530    ----VRH--DAAGAKAWVT-------EARA--DGETCMDECRM----TEG------GA-APEIADRIDELA--------KLCSIA-----L-------ALTNASMSKHP-----------
Os01g20970    -----TG--GYEEAMALVA-------AGQA--EVKMCQRGFKA-----VP------QH-RNILTLRNREVD--------QLCSIA-----F-------TITKLIRVSPSAEE--------
Os05g29740    -----AG--EFDEAELLVG-------AGRT--EAELCQKGCEH-----AR------LP-AL-LAARNGAVE--------RLCNVA-----M-------DITRLLHQQH-----------
Os02g01300    -----SW--RYGDAKTAMS-------ATTD--APVTCEDEFKG-----QG------MATPPAIKARTKPLF--------QHGVIS-----L-------AIISLL--------------
Os03g40900    ------------------------------GVERAVAGPEV-------------------LWRVRDAQT---------------------------------------------------
Os08g12600    AGDGKRSKEDLENVQKWIKGVDKHYVGGNA--AGSDCETGYL--------------YTYNDDLPAQETLGN--------CLYTAS-------------SLINHIKL-------------
Os08g12160    AGNGKRSKEDLENAQKWIKGVEKPYNGGIG--KASGCEIGYL--------------FTYSDDLPAQKTLGY--------TFDTAS-------------SLINHIKL-------------
Os08g12230    -----------------------------------------------------------------------------------------------------------------------
Os08g12520    AGEGKRSKEDLETVHKWTK-------NLET--QYDETASKCKLGDLFKYG------DKKVPTVWVIDLLTT--------TAI-----------------RLLNAIKP-------------
Os08g19780    -----KGRRKKEDVETVLKWTKNLETQYNG--ATSKCKLGYL--------------FKYCD-MVPTVKEID--------AATTIT-----I-------DLLNAIKL-------------
Os03g43820    RGSEEQRASDKLRANVWLT-------SAMD--SGVSCTDWFNE-----EG------SHGRPASSPVGKKVI--------AGCATATQYMSI-------ALELLVNCITT-----------
Os10g10700    KGSQARW--AVSNAKTWLS-------AAMT--NEANCADALSSTGAAVSP------AA-RE-LIAGVVMAK--------QYTSIA-----L-------SFVNTIPVS-----------
Os10g10630    GSRLARW--EVSNAKTWLS-------AAMA--NVATCADGFADADSWSAA------GI-EEVVAGEAANVS--------KYTSNA-----L-------ALVNGIPL------------
Os10g10560    GSRQARW--QLSNAKTWLS-------AAMT--NEGTCSDGFEDAGAAATA------SPAGKEVAAGVASVT--------QHTSNA-----L-------ALVNGIPL------------
Os10g10620    SSRQARW--HVSNVQTWLS-------AAIT--DEGTCTDGFEEAGEAAAG------SPAGKEVAAGVARVK--------QHTSIA-----L-------ALVNGIPL------------
Os10g36500    AAARRKVRFEVDNVRTWAS-------AALT--DDNMCMEGFKGEAAGGGG------AR-EA-VRGHIMGLL--------HLTANA-----L-------GILNAMAKQI----------
Os04g01570    GSPRFAW--RLSNVQTWAS-------AALT--DASTCLDSLAT--YAAPG------ID-VDALRKRVGAVS--------QATSNA-----L-------ALVNNLDPHHHL---------
Os06g49760    GSPGFKW--HLSNVQTWCS-------AALT--DENTCLDGLSR----GVD------AATRSAIRGKIVEVA--------QVTSNA-----L-------ALVNRVAPAN----------
Os08g24160    GSPGFKW--HLSNVQTWCS-------AALT--DKNTCLDGLSRGVDAATR------SATRGKIVVGVHRRRPPSSSTAWRGCGGR-------------RLLVAGRARTRA---------
Os12g37480    -----AG--DKDTTGTMLD-------TART--DVDTCDQGFEE----RDG------DI-PPLMSKQDAELA--------KLASNC-----I-------AIAVAAGLRSSS---------
Os08g01670    -----QQ--DYDLATVHVS-------AGAE--YPQVCKALFRR----QRP------GAYPAELAAREEALN--------KLCSVA-----L-------DIIALLTSSPASNNNNS-----
Os12g18560    -----QQ--DYDMASVHVS-------AAAE--YPQVCRTLFGR----QSP------GDYPPELAATEVALR--------QLCSVA-----L-------DIIALLSSSSN----------
Os02g46290    ----VGR--PAVTSFYKLH-------RASD--EPGNCDIMLFE-----GS------AHKNP-VSSENMYNE--------AISKLT-----S-------DIVYQLVH-------------
Os02g01310    -----VR--IYGTAMEQLS-------AALG--AAESCEDAWKG-------------DEGNIPIAAHDREYG--------RMAHIA-----I-------GFTHAAA-------------
Os05g05640    -----SR--RYAAARQELA-------RVAA--LVQRCDGGLSR-----AG------AR-SP-LPRYSADCQ--------QVAIMG-----I-------AITNLLK-------------
Os03g01020    -----NR--AYDVGEKKLD-------EALS--LTQKCNAAFAK---IGVP------LQ-QP-LAQLTADTI--------QIAIIAKAITCLVNVNNNPALVAAAAAAAAAKAPQQSQYP-
Os02g33130    -----AQ--SYAAGKEKVG-------EAIP--LARQCDDAFAK-----AA------VP-SP-LVQRSWCSV--------QMSIIC-----T-------AITNLIK-------------
Os02g33380    -----RR--EYAAGKEKAG-------EAAS--LARRCDGAFAD-----AG------VAPSP-LERQTAESV--------KIAIVC-----T-------AITNLVK-------------
Os02g33390    -----AR--DYAAGKGKAA-------DAAS--LARQCDAAFAG--KDDAA------AAVPPVIAQHGSYAA--------RIAIVC-----T-------AITNLIE-------------
Os08g42890    -----GG--AYLDVVRHQL-------AGYY--AGIMCDNMIVR-----RS------KV-SP-VADIDRTTA--------THCNIA-----V-------DLIGLLY-------------
Os01g02070    -----SG--DYAAASRLVS-------GASG--AADTCDAAFAA-----AK------VS-SV-MVDVDQKMK--------DRCGVA-----R-------DLINLLIKFPPSDN--------
```

(**C**)

**Figure S4**. Comparison of expression of all cell wall invertases during grain development using RMA normalized data from Expression data for reproductive development in rice [20] accessed as accession OS5 from PLEXdb. Data represent the mean of 3 samples ± standard error.

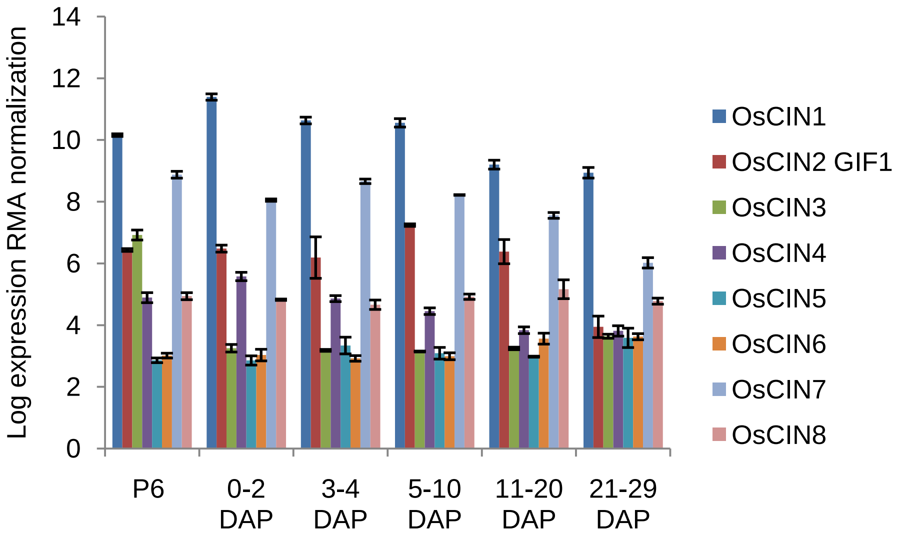


**Table S1.** Genes with a pairwise mutual coexpression rank <50 with each of the bait genes, *OsINVINH3*, *OsIAA29* and *OsYUC12*. Pearson correlation coefficients (PCC) are shown. Genes listed in order of increasing mutual co-expression rank. Cysteine rich proteins are coloured yellow, ZmEBE homologues are coloured red, INVINH/PMEI homologues are coloured green, putative transcriptional regulators are coloured blue.

| Transcript ID | PCC *OsYUC12* | PCC *OsIAA29* | PCC *OsINVINH3* | Product Description |
|---|---|---|---|---|
| Os12g13960.1 | 0.8738 | 0.9242 | 0.9658 | LTPL33 [37] |
| Os02g07628.1 | 0.8669 | 0.9171 | 0.9448 | DEF5 [37] |
| Os11g45360.1 | 0.8901 | 0.8848 | 0.9293 | DEFL15 [37] |
| Os01g70680.1 | 0.8909 | 0.8474 | 0.9399 | DEF1 [37] |
| Os02g07624.1 | 0.8217 | 0.9411 | 0.9280 | DEF4 [37] |
| Os11g18140.1 | 0.8363 | 0.9109 | 0.9132 | ZmEBE 1 homologue |
| Os04g49720.1 | 0.8391 | 0.8957 | 1.0000 | OsINVINH3 |
| Os06g17480.1 | 0.8499 | 0.8796 | 0.9102 | OsHAP3D [34] |
| Os05g07850.1 | 0.8366 | 0.8900 | 0.9073 | Leucine-rich repeat receptor-like kinase [39] |
| Os11g14880.1 | 0.8300 | 0.8920 | 0.9009 | LTPL32 [37] |
| Os11g11430.1 | 0.8189 | 1.0000 | 0.8957 | OsIAA29 [40] |
| Os01g28810.1 | 0.8250 | 0.9185 | 0.8727 | expressed protein (nuclear) |
| Os04g12639.1 | 0.7898 | 0.9329 | 0.8954 | ZmEBE 1 homologue |
| Os08g35670.1 | 0.8177 | 0.8742 | 0.8963 | OsRR33 [35] |
| Os12g12230.1 | 0.8682 | 0.8561 | 0.8678 | DEFL43 [37] |
| Os02g15710.1 | 0.8613 | 0.8141 | 0.8996 | plastocyanin like (extracellular) |
| Os08g16650.1 | 0.7807 | 0.9250 | 0.8677 | hypothetical protein (nuclear) |
| Os02g54960.1 | 0.7439 | 0.9141 | 0.8948 | ZmEBE 1 homologue |
| Os02g07550.1 | 0.7717 | 0.8823 | 0.8725 | DEFL1 [37] |

**Table S1.** *Cont.*

| **Transcript ID** | **PCC *OsYUC12*** | **PCC *OsIAA29*** | **PCC *OsINVINH3*** | **Product Description** |
|---|---|---|---|---|
| Os09g11790.1 | 0.8456 | 0.8099 | 0.8935 | DEFL14 [37] |
| Os11g03870.1 | 0.8439 | 0.8172 | 0.8885 | LTPL31 [37] |
| Os01g26320.1 | 0.8027 | 0.8249 | 0.8926 | ZmEBE 1 homologue |
| Os08g42890.1 | 0.7936 | 0.8257 | 0.9268 | INVINH/PMEI homologue |
| Os01g12030.1 | 0.8342 | 0.8226 | 0.8387 | endoglucanase GH9 (plasma membrane) |
| Os01g67800.1 | 0.7783 | 0.8638 | 0.8234 | retrotransposon protein, putative, |
| Os12g27940.1 | 0.8283 | 0.8332 | 0.8282 | low glutelin content-like (extracellular) |
| Os02g17230.1 | 1.0000 | 0.8189 | 0.8391 | OsYUC12 |
| Os02g07600.1 | 0.7703 | 0.8390 | 0.8661 | DEF3 [37] |
| Os03g39170.1 | 0.7341 | 0.8901 | 0.8410 | frigida, putative, expressed |
| Os05g48560.1 | 0.7671 | 0.8302 | 0.8339 | expressed protein |
| Os10g19925.1 | 0.7100 | 0.8584 | 0.8487 | DEFL68 [37] |
| Os08g04740.1 | 0.7645 | 0.8646 | 0.8193 | INVINH/PMEI homologue |
| Os11g41880.1 | 0.8008 | 0.7907 | 0.8628 | Homologue of OBERON1/OBERON2 nuclear PHD finger proteins |
| Os02g38940.1 | 0.7604 | 0.8958 | 0.8108 | cytochrome P450 71D8 (P450 CP7) |
| Os11g11530.1 | 0.7693 | 0.8206 | 0.8549 | ZmEBE 1 homologue |
| Os07g41290.1 | 0.7223 | 0.8968 | 0.8302 | DEFL13 [37] |

**Table S2.** Primers used for qRT-PCR.

| **Gene ID** | **L primer** | **Tm ℃** | **R primer** | **Tm ℃** | **Product size** |
|---|---|---|---|---|---|
| Os02g42314 | CTGCGAGCTGAAACACTTTG | 59.78 | TTCTCGCTGCACCTCCTTAT | 59.98 | 105 |
| Os04g49720 | GGCCATCGACTACCCCTACT | 60.35 | CCGCCTCGGTGATCCT | 60.76 | 107 |
| Os11g11430 | CAACTATTCCATGTCACCATTTG | 59.26 | ATGGGACATCACCAAGGAAG | 59.78 | 131 |
| Os02g17230 | GAGTTATCGACGTGCTCGAA | 59.03 | TGCTTTCACCATTCTTTAGCC | 59.35 | 147 |
| Os07g41290 | TTGCTACGCACATGTTGAGG | 60.87 | TGCCTGCAATACTGAGATGC | 59.98 | 123 |
| Os11g14880 | CTCATCGCCAGGTTTTGG | 60.19 | GATGCCAGGCTGATCAATTT | 60.04 | 103 |
| Os11g18140 | AGATCGCGTGGGTTGATAAT | 59.42 | ACAGTGGAAACTGGCCTGA | 59.25 | 112 |
| Os06g17480 | GTCGGCCCCAACTATGAGTA | 59.96 | TGCTAATCGATCGACAGACG | 59.97 | 115 |
